# Identification and validation of diagnostic genes *IFI44* and *IRF9* in insomnia-associated autoimmune uveitis

**DOI:** 10.3389/fimmu.2025.1519371

**Published:** 2025-01-31

**Authors:** Chao Wu, Hui Feng, Meng Tian, Baorui Chu, Xianyang Liu, Shuhao Zeng, Yakun Wang, Hong Wang, Shengping Hou, Qingfeng Liang

**Affiliations:** ^1^ Beijing Institute of Ophthalmology, Beijing Tongren Hospital, Capital Medical University, Beijing, China; ^2^ Department of Ophthalmology, Qilu Hospital, Shandong University, Jinan, China; ^3^ The First Affiliated Hospital of Chongqing Medical University, Chongqing, China

**Keywords:** insomnia, autoimmune uveitis, IFI44, IRF9, CMAP

## Abstract

**Objective:**

Insomnia is increasingly recognized as a significant factor in the development of various autoimmune diseases, including autoimmune uveitis (AU). We investigated insomnia-associated genes that may contribute to AU pathogenesis and sought to identify potential biomarkers for insomnia-associated AU.

**Methods:**

Microarray data related to insomnia and AU were downloaded from the Gene Expression Omnibus (GEO) database and analyzed. The GEO2R tool was used to identify differentially expressed genes (DEGs) that were common between insomnia and AU. Weighted gene co-expression network analysis (WGCNA), protein-protein interaction (PPI), functional enrichment, and CMap analyses were then performed to identify pathogenic genes, underlying mechanisms, and potential therapeutic drugs for insomnia-associated AU. Least absolute shrinkage and selection operator regression was employed to screen for candidate biomarkers, and their diagnostic performance was evaluated using receiver operating characteristic (ROC) curves and quantitative polymerase chain reaction (qPCR). Finally, molecular docking was applied to verify binding activities.

**Results:**

We identified 138 up-regulated and 85 down-regulated DEGs that were common to insomnia and AU. PPI network analysis highlighted 10 key genes, CMap analysis identified 30 compounds, and WGCNA revealed 54 key genes and 30 compounds. Intersection of the above-mentioned key genes and compounds identified six genes and five compounds. After verification by qPCR and ROC curve analysis, *IFI44* and *IRF9* were confirmed as hub genes. Finally, two compounds were selected based on docking scores of less than −7 kcal/mol.

**Conclusion:**

Our study demonstrated involvement of the viral response in both insomnia and AU and identified the diagnostic significance of *IFI44* and *IRF9* in these conditions. These findings provide novel insights for future diagnostic and therapeutic strategies to treat insomnia-associated AU.

## 1 Introduction

Insomnia disorder is the second most prevalent mental disorder (1) and the most common sleep complaint, affecting up to one-third of the adult population worldwide (2). Insomnia is defined as difficulty initiating or maintaining sleep despite adequate sleep opportunities and a conducive environment, which sufferers perceive to negatively impact daytime functioning. The diagnostic criteria for insomnia are when the symptoms occur at least three times per week and persist for at least three months (2). Although the mechanisms underlying sleep disorders are not fully understood, evidence that has accumulated over the past few decades implicates immune-related molecules in sleep regulation (3–6). Moreover, sleep disruption can activate inflammation, creating a positive feedback loop that exacerbates the condition. Sleep disorders have been observed in some autoimmune diseases, such as rheumatoid arthritis, systemic lupus erythematosus, and Behçet’s disease (7).

Autoimmune uveitis (AU) is a prevalent immune disorder that can cause blindness. Patients frequently experience significant intraocular inflammation and various systemic symptoms, including those associated with Behçet’s disease, Vogt-Koyanagi-Harada disease, seronegative spondyloarthropathies, and multiple sclerosis (8, 9). The recurrent nature of the disease can lead to severe damage to the retina and optic nerve. The pathogenesis of AU involves disruption of the blood–retinal barrier and the activation of Th17 cells and microglia (10, 11). Epidemiological evidence indicates that uveitis accounts for approximately 25% of blindness in developing countries (12). Current treatments for AU typically include corticosteroids, novel immunosuppressants, and antimetabolite drugs. However, these treatments are suboptimal because of the complex etiology and strong heterogeneity of AU (13). Consequently, AU patients remain at risk of vision loss despite available therapeutic interventions.

A meta-analysis showed that patients with Behçet’s disease have poorer sleep quality compared with the general population, which was attributed to changes in sleep parameters and a higher incidence of specific sleep disorders (14). Behçet’s disease is a chronic systemic vasculitis affecting small to medium vessels, and is characterized by a relapsing-remitting course, and most frequently by mucocutaneous, ocular, and articular involvement (15). A case control study revealed that sleep duration of less than 7 hours/day (OR 12.12, 95% CI 1.37–107.17, p = 0.025) is a risk factor for uveitis onset (16). Stress and sleep deprivation can trigger uveitis flare-ups in patients with idiopathic recurrent acute anterior uveitis. Those patients with insufficient sleep had an approximately 12 times higher chance of a uveitis flare-up in the following month. Additionally, sleep deprivation can promote Th17 cell pathogenicity and AU onset (17). However, the mechanisms by which sleep deprivation affects uveitis onset or recurrence remain unclear. Further exploration of gene expression in insomnia-associated AU is crucial to understanding the impact of insomnia on uveitis onset and recurrence.

This study analyzed microarray data from the Gene Expression Omnibus (GEO) database, specifically focusing on peripheral blood mononuclear cells (PBMCs) from patients with insomnia or autoimmune uveitis (AU) (datasets GSE208668 and GSE66936). Potential therapeutic small molecule compounds were identified using the CMap database and validated via molecular docking. These findings offer new insights into the pathogenesis of both uveitis and insomnia.

## 2 Material and methods

### 2.1 GEO dataset processing

We searched the GEO database for gene expression profiles related to “insomnia” and “autoimmune uveitis”. The obtained datasets were filtered based on the following criteria: (1) the gene expression profiles must include samples from patients with insomnia, uveitis, and controls. (2) The sequencing data must be obtained from PBMCs. Based on these criteria, the GEO datasets, GSE66936 and GSE208668, were selected. **Table 1** provides a detailed overview of these datasets.

**Table 1:** Information of GEO datasets involved in this study.

GSE number	Platform	Samples	Source	Disease
GSE66936	GPL570	4 patients VS 17 controls	PBMCs	autoimmune uveitis
GSE208668	GPL10904	17 patients VS 25 controls	PBMCs	insomnia

### 2.2 Identification of differentially expressed genes common to insomnia and AU

GEO2R (https://www.ncbi.nlm.nih.gov/geo/geo2r), an official tool of the GEO database that uses linear models for microarray analysis, was employed to compare samples and to identify differentially expressed genes (DEGs) across experimental conditions in GSE66936 and GSE208668 datasets. This analysis aimed to reveal common genetic characteristics between insomnia and uveitis. The Benjamini-Hochberg false discovery rate method was applied, with an adjusted P value < 0.05 and |log2 FC| > 0.58 as the threshold for DEG screening.

### 2.3 Weighted gene co-expression network analysis

WGCNA, an algorithm widely used to find co-expression gene modules with high biological significance, was employed to explore the relationship between screened gene networks and diseases using the Spearman correlation coefficient. The analysis was conducted using ImageGP (http://www.ehbio.com/ImageGP), a web application based on a high-level web framework for backend data preprocessing and analysis, primarily based on the R programming language. To avoid redundant modules, we adjusted the parameters as follows: the minimum module size was set to 10 for insomnia and 100 for uveitis, with a deep split of 4. Other parameters were network type = “signed” and R square cut = 0.85. Finally, the expression profiles of each module were summarized by the module eigengene, and the correlation between the module eigengene and clinical features was calculated. Modules with a high correlation coefficient with clinical features were selected for subsequent analyses.

### 2.4 Protein–protein interaction network construction

PPI networks were constructed using STRING (https://string-db.org/) and visualized using the Cytoscape platform. Significant modules and core genes were identified using the Cytoscape plugins, CytoHubba and MCODE. Two different algorithms, Maximal Clique Centrality, and Density of Maximum Neighborhood Component, were used to identify hub genes.

### 2.5 LASSO regression analysis

The hub genes were further identified using LASSO analysis. LASSO analysis is a regression method that improves prediction accuracy by selecting a variable from high-dimensional data with strong predictive value and low correlation. A LASSO logistic regression model was then built based on the expression levels of these hub genes and clinical traits.

### 2.6 Small molecule compound screening and molecular ligand docking analysis

The Connectivity Map database (CMap, https://clue.io/) is a differential gene expression-based drug prediction database, primarily used to explore the functional relationships among genes, small molecule compounds, and diseases. The primary protein structures of the target genes were downloaded from The Protein Data Bank database (http://www.rcsb.org, PDB). AutoDock Tools software (version 1.5.7) was used to perform molecular docking of the key targets with small molecule compounds. The binding activities of these compounds to their targets were evaluated based on docking energy values using Pymol software (http://www.pymol.org).

### 2.7 Real-time quantitative polymerase chain reaction

Peripheral blood was collected from all subjects into tubes containing ethylene diamine tetraacetic acid. PBMCs were isolated by centrifugation through a Ficoll-Paque (Sigma-Aldrich) density gradient. Total RNA from PBMCs was extracted using TRizol reagent (Thermo Fisher Scientific, USA), and RNA concentration was assessed by Nanodrop 2000 spectrophotometry (Thermo Fisher Scientific). Total RNA was reverse transcribed into cDNA using HiScript III All-in-one RT SuperMix Perfect (Vazyme Biotech Co.). Real-time PCR quantification (RT-qPCR) was performed on a QuantStudio 3 Real-Time PCR System (Thermo Fisher Scientific) using ChamQ Universal SYBR qPCR Master Mix (Vazyme Biotech Co.). RT-qPCR primer sequences were: *IFI44* forward: 5′-TGTGAGCCTGTGAGGTCCAAG-3′, *IFI44* reverse: 5′-AATTGCTAACCACCGAGATGTCAG-3′; *IRF9* forward: 5′-CTGCTGCTCACCTTCATCTACAAC-3′, *IRF9* reverse 5′-ACCTGCTCCATGCTGCTCTC-3′; *β-actin*: forward: 5′-CCACGAAACTACCTTCAACTCCATC-3′, reverse 5′-AGTGATCTCCTTCTGCATCCTGTC-3′.

### 2.8 Statistical analysis

LASSO regression was performed using the glmnet R package. All statistical tests were two-tailed, with P < 0.05 considered statistically significant.

## 3 Results

### 3.1 DEGs common to insomnia and uveitis

**Figure 1:**
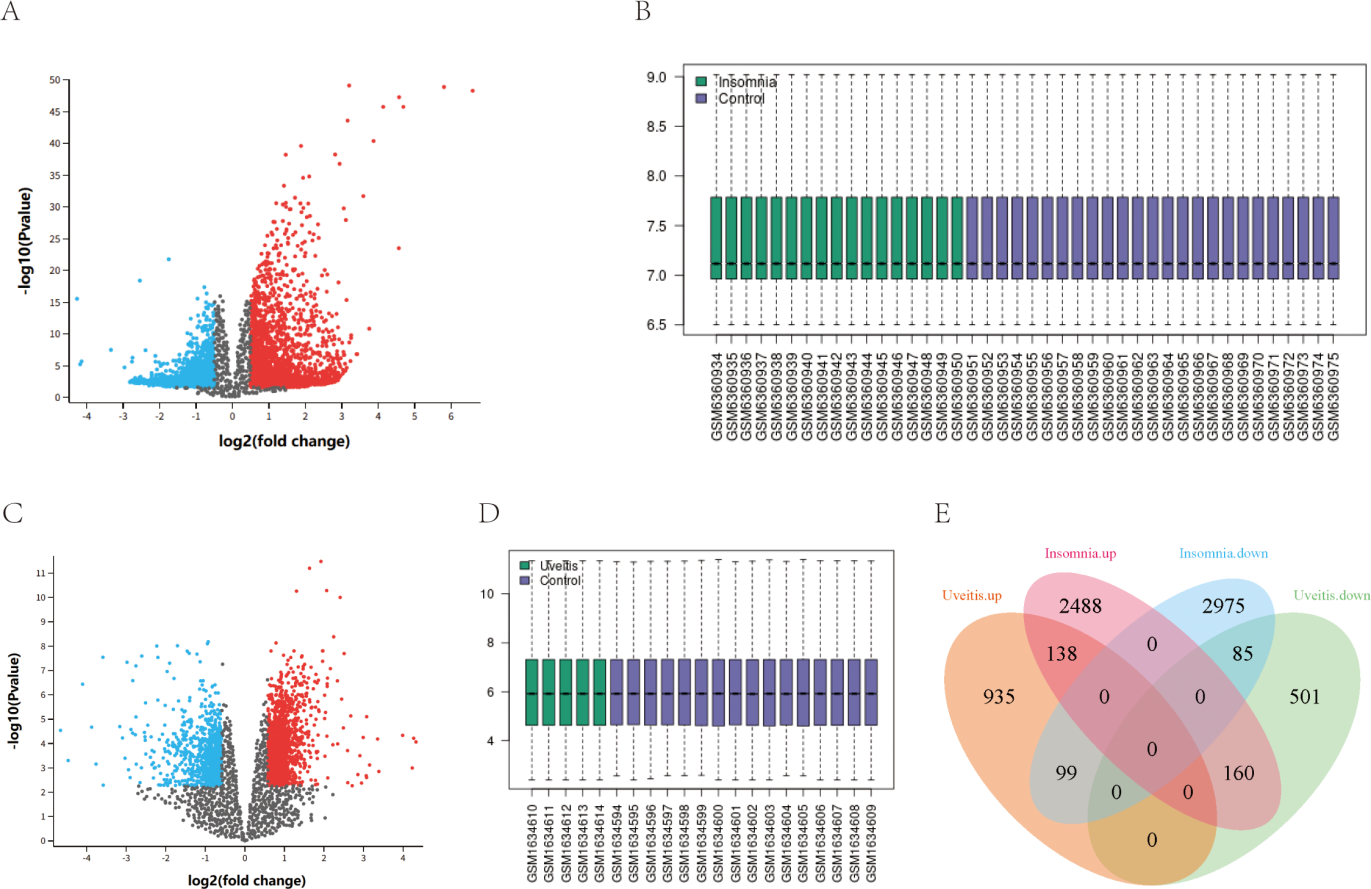
The identification of shared differentially expressed genes. **(A, C)** Volcano map of DEGs in GSE208668 and GSE66936. **(B, D)** normalized expression matrices in GSE208668and GSE66936. **(E)** The shared DEGs of the Uveitis dataset and Insomnia dataset are represented by a Venn diagram.

Analysis of differential gene expression between insomnia and control samples revealed 5945 DEGs, with 2786 up-regulated and 3159 down-regulated. Similarly, 1172 up-regulated and 746 down-regulated DEGs were identified between uveitis and control samples. Among these, 138 up-regulated and 85 down-regulated DEGs were common between insomnia and uveitis (**Figure 1**).

### 3.2 Screen for key genes in insomnia and uveitis

**Figure 2:**
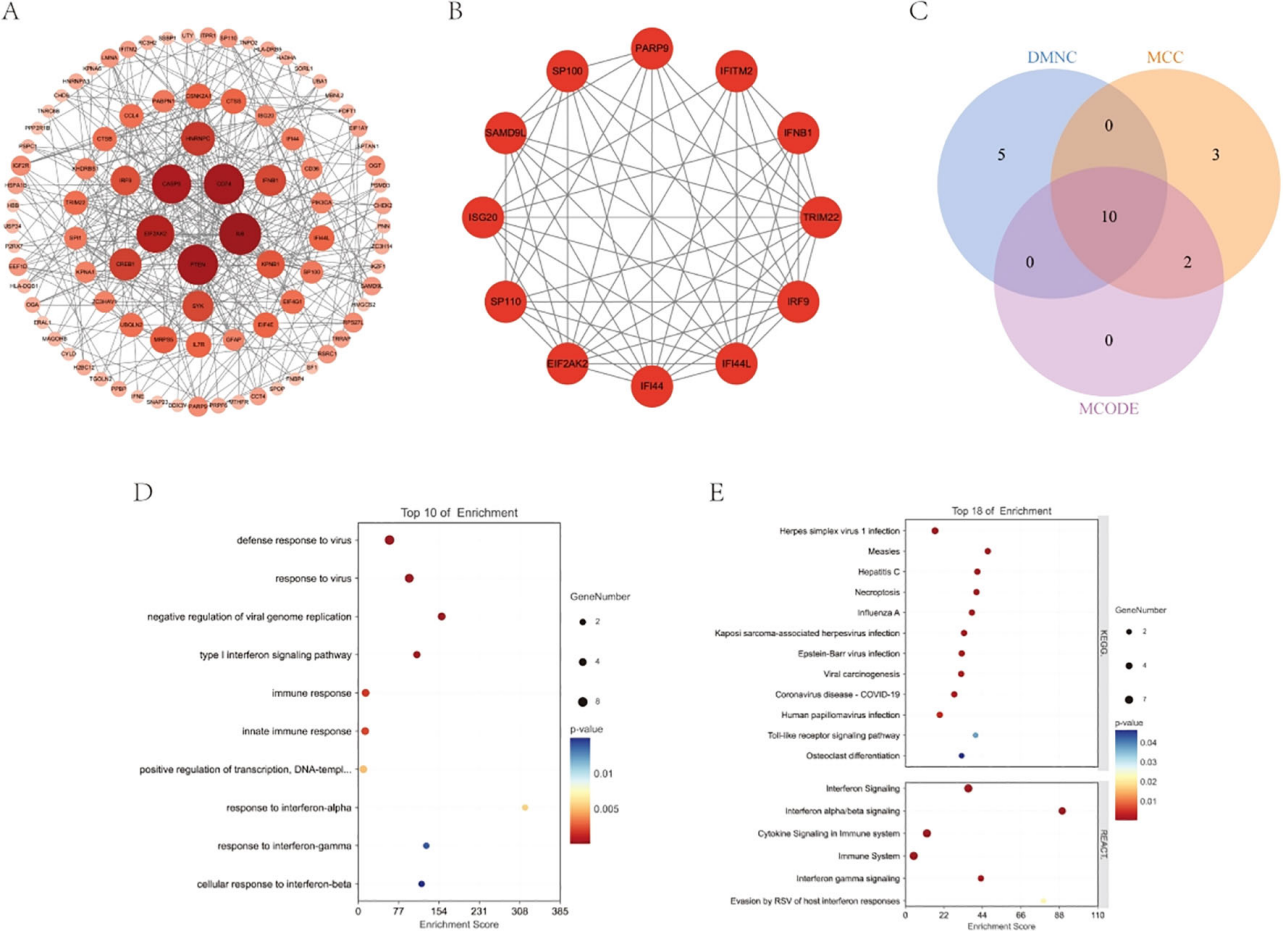
PPI analysis network construction and enrichment analysis. **(A)** The PPI network of shared differentially expressed genes. **(B)** The PPI network of module 1 genes with the top 1 highest score based on MCODE analysis. **(C)** Venn diagram showed the overlapping genes between MCODE, MCC and DNMC analysis. **(D, E)** The bubble plots showing the GO, KEGG and REACTOME enrichment analysis.

STRING was used to generate PPI networks of shared DEGs to clarify their interactions (**Figure 2A**). The CytoHubba methods, Maximal Clique Centrality, and Density of Maximum Neighborhood Component, were used to predict and explore the top 15 key genes in the PPI network. Ten key genes that were up regulated in both diseases were identified: *ISG20*, *SAMD9L*, *IFI44*, *IFITM2*, *PARP9*, *IRF9*, *SP100*, *IFI44L*, *TRIM22*, and *SP110*. Subsequently, six cluster modules were identified using the MCODE plugin, with cluster 1 having the highest score (score of 10, 12 nodes, and 55 edges). The above 10 genes were consistent with this cluster (**Figure 2B**). Moreover, pathway enrichment analyses were conducted on these 10 key genes. Gene Ontology enrichment analysis indicated that these genes were significantly involved in the response to viruses, including defense response to viruses, negative regulation of viral genome replication, and the type I interferon signaling pathway (**Figure 2C**). Kyoto Encyclopedia of Genes and Genomes enrichment analysis also revealed associations with virus infection. Reactome enrichment analysis revealed the activation of multiple virus response-related pathways, including interferon, interferon alpha/beta, and interferon gamma signaling pathways (**Figure 2D, E**).

**Figure 3:**
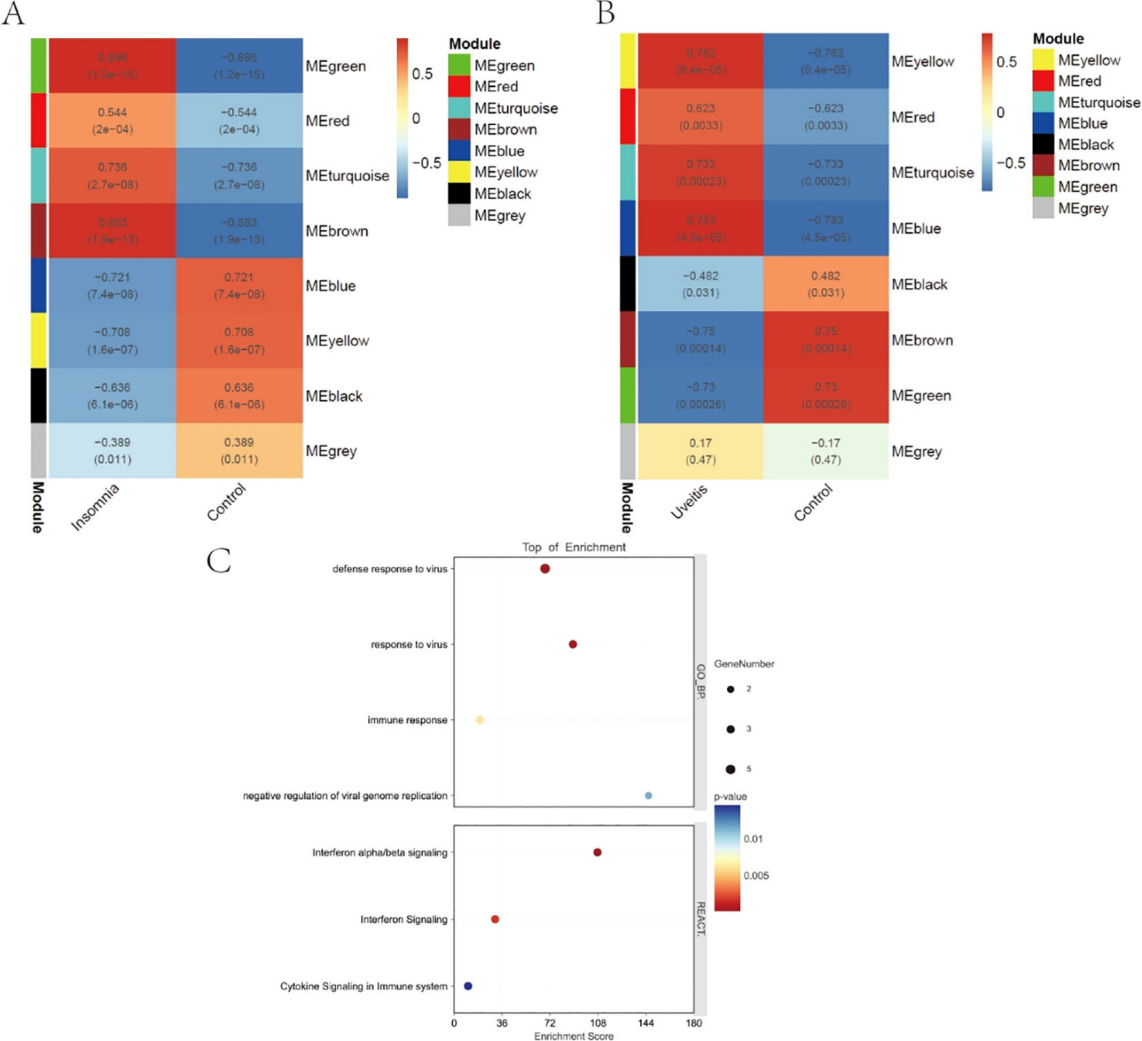
WGCNA and enrichment analysis. **(A, B)** Weighted gene co-expression network analyses of insomnia and uveitis. **(C)** The bubble plots showing the GO and REACTOME enrichment analysis of final 5 key genes.

Eight modules were identified in both insomnia and uveitis (**Figures 3A, B**). Heatmaps illustrating module–trait relationships were used to evaluate the associations between each module and the diseases. To study the pathogenic genes, we focused only on the modules that were positively correlated with the traits. Specifically, the turquoise, brown, and green modules in insomnia and the turquoise, yellow, and blue modules in uveitis were selected for further analysis, based on Spearman’s rank correlation coefficient, which exceeded 0.7 (P < 0.05). Intersection of uveitis and insomnia genes using a Venn diagram revealed 54 genes. Comparison of these with previously identified key hub genes identified five common genes: *ISG20*, *IFI44*, *IFITM2*, *IRF9* and *IFI44L*. Pathway enrichment analyses were then performed and, consistent with the PPI findings, Gene Ontology enrichment revealed significant involvement of these genes in response to virus, while Reactome enrichment analysis indicated the activation of multiple virus response-related pathways (**Figure 3C**).

### 3.3 The validation of hub genes in insomnia and uveitis

**Figure 4:**
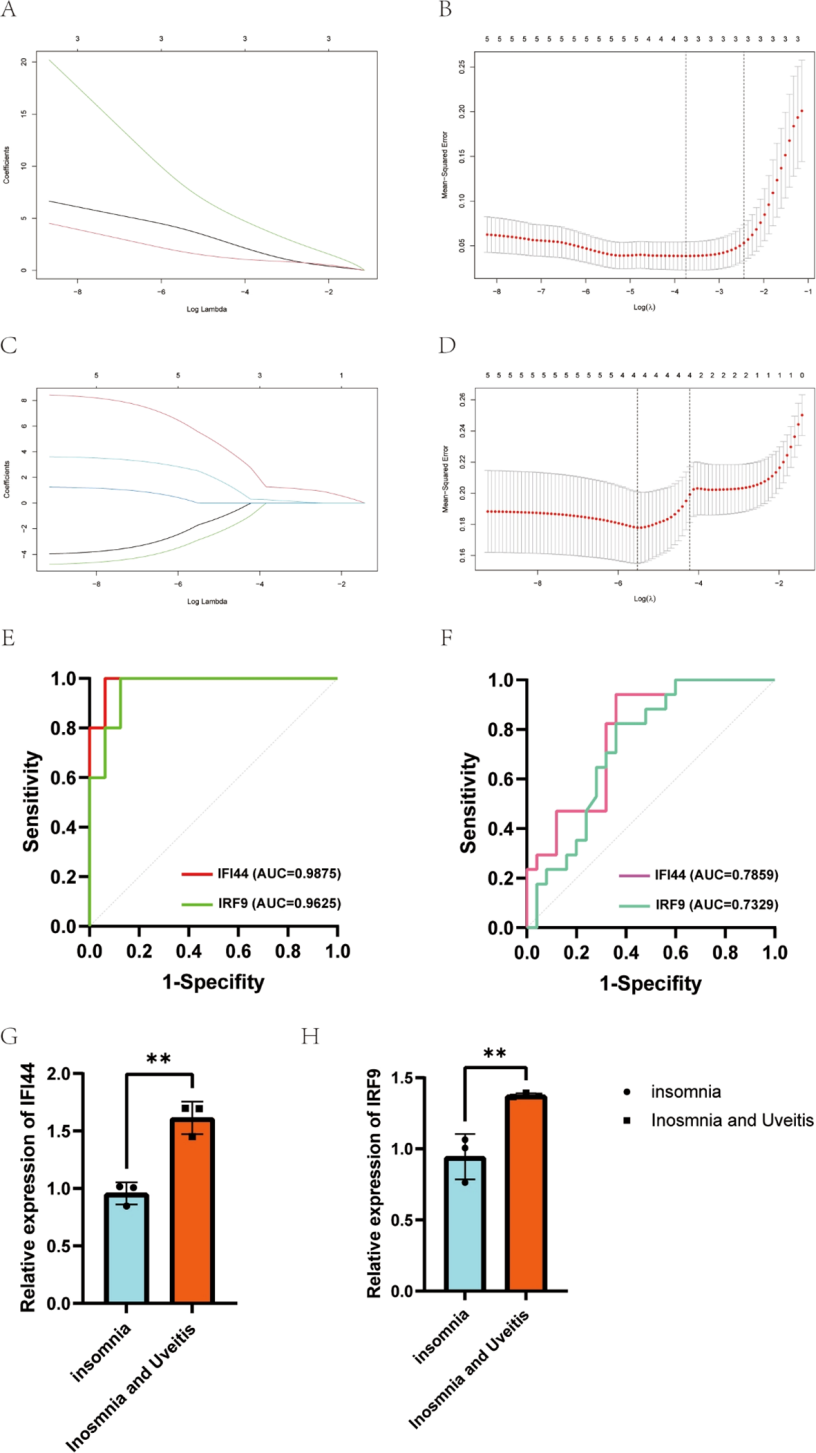
Candidate genes were screened in the Lasso model and ROC curve analysis. **(A, B)** 3 potential biomarkers were identified by LASSO regression in uveitis. **(C, D)** 4 potential biomarkers were identified by LASSO regression in insomnia. **(E, F)** The diagnostic accuracy of hub genes for uveitis **(E)** or insomnia **(F)** was evaluated by ROC curves analysis. **(G, H)** The identification of expression of *IFI44* and *IRF9* between insomnia and insomnia with uveitis from PBMCs.

LASSO regression was applied and identified three candidate hub genes out of five key genes with significant potential for diagnosing uveitis (**Figures 4A, B**). Subsequently, four potential hub genes out of these five candidate hub genes were found to have a strong impact on diagnosing uveitis (**Figures 4C, D**). Among these, *IFI44*, *IFI44L*, and *IRF9*, were identified as having a significant effect on diagnosing insomnia-associated uveitis. According to ROC curve analysis, the area under the curve of *IFI44* and *IRF9* was greater than 0.7 in both insomnia and uveitis (**Figures 4E, F**). Finally, *IFI44* and *IRF9* expressions in PBMCs from subjects with insomnia with or without uveitis were confirmed by qPCR (**Figures 4G, H**).

### 3.4 Prediction of potential drugs for patients with insomnia and uveitis

**Figure 5:**
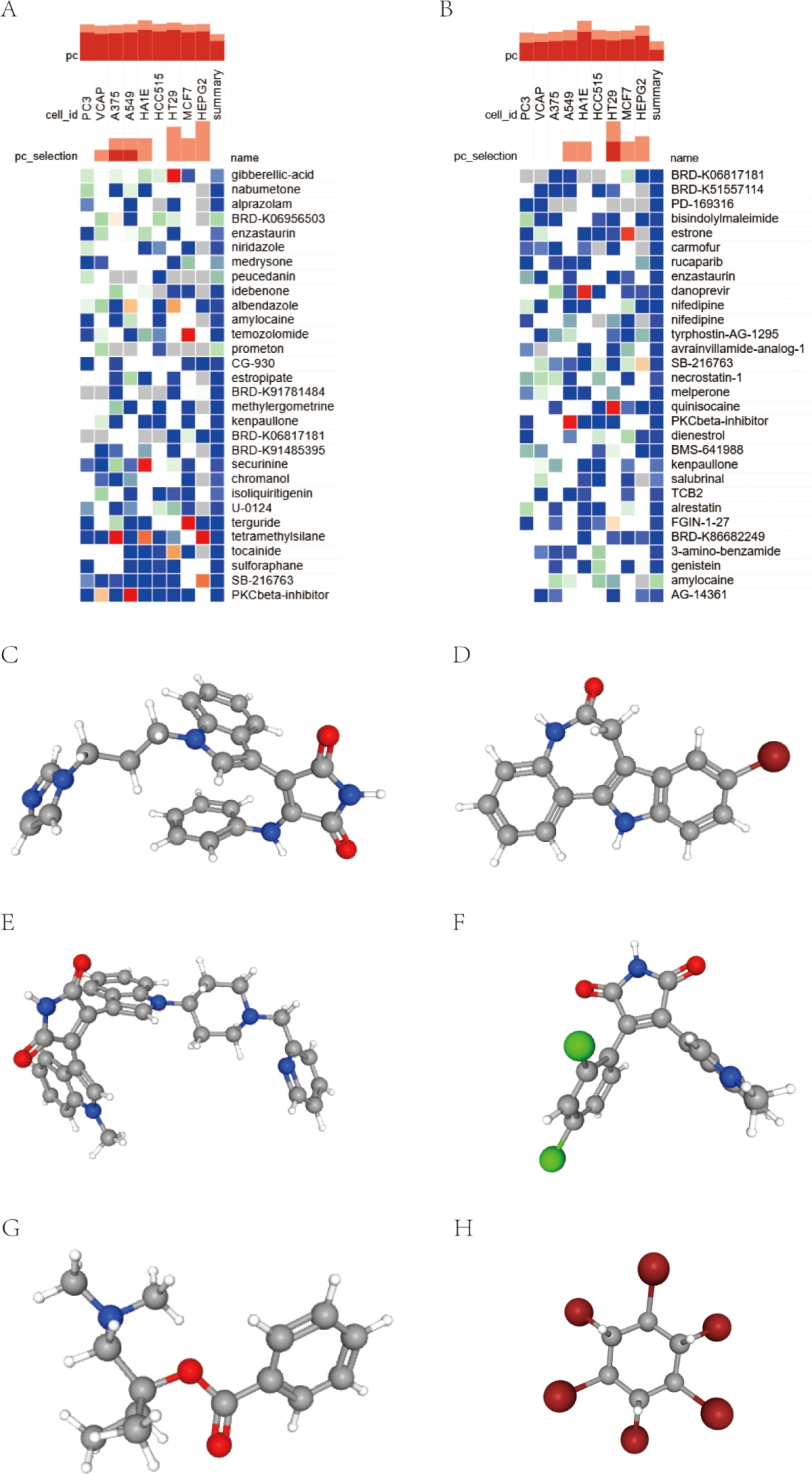
Screening of the compounds for the treatment of insomnia with uveitis by CMap analysis. **(A, B)** The heatmap presenting the top 30 compounds with the most significantly negative enrichment scores in 10 cell lines based on CMap analysis. **(C–G)** The chemical structures of 6 compounds from the intersection of above two 30 compounds were shown.

We submitted the 10 and 54 genes described above to the CMap database to screen for small molecule compounds that have potential efficacy in the management of insomnia-associated uveitis. After intersection, the top six compounds, with the highest negative scores, were identified as potential therapeutic agents: PKCbeta-inhibitor (BRD-K89687904), kenpaullone, enzastaurin, SB-216763, amylocaine, and BRD-K06817181 were potential therapeutic agents for the treatment of insomnia-associated uveitis (**Figures 5A, B**). The structures of these compounds were retrieved from the PubChem database and are displayed in **Figures 5C–H**.

### 3.5 The molecular docking of two compounds against insomnia-associated uveitis

**Figure 6:**
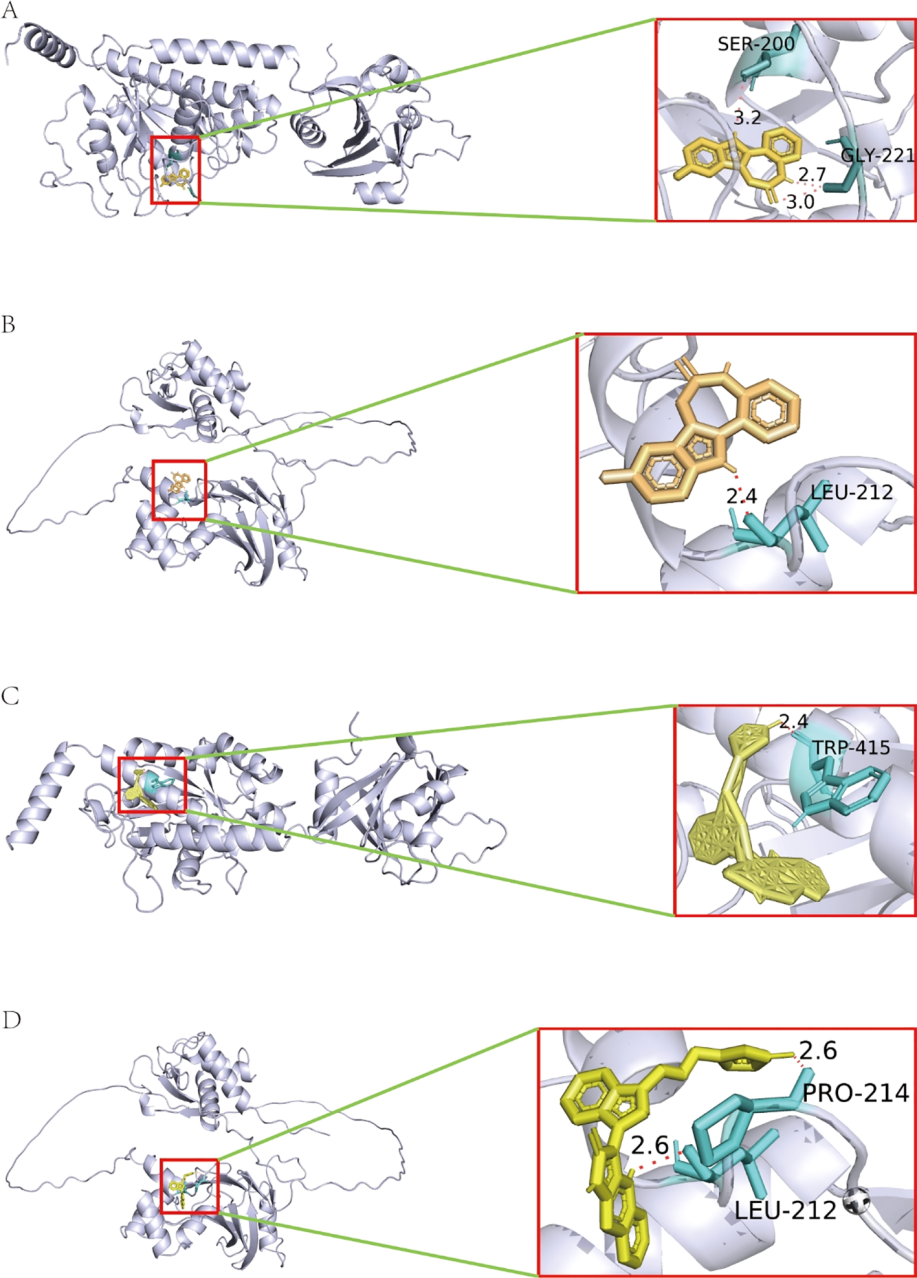
Molecular docking analysis. **(A, B)** Molecular docking pattern of PKC beta Inhibitor complexed with IFI44 and IRF9. **(C, D)** Molecular docking pattern of kenpaullone complexed with IFI44 and IRF9.

Molecular docking is an important method for structure-based drug design and screening that finds the optimal conformation of small molecule compounds and target molecules for interaction. In this study, the crystal structures of two molecular targets, IFI44 (ID: Q8TCB0) and IRF9 (ID: Q00978), were downloaded from the AlphaFold Protein Structure Database. We used AutoDock Tools 1.57 software to dock the above five compounds that have treatment potential for insomnia-associated uveitis on the IFI44 and IRF9 molecular targets. The docking scores of PKCbeta Inhibitor and kenpaullone were less than −7 kcal/mol, indicating a high binding affinity of both compounds with the targets. The binding poses and sites are shown in **Figures 6A–D**, where the yellow color represents the compounds, and the red dotted lines represent hydrogen bond interactions.

## 4 Discussion

In this study, we used bioinformatic analyses to identify potential diagnostic genes for insomnia-associated uveitis. We then validated these findings using PBMCs from participants and identified *IFI44* and *IRF9* as key genes. Additionally, we explored potential therapeutic drugs for insomnia-associated uveitis using the CMap database. Finally, molecular docking identified two small molecule compounds that target IFI44 and IRF9.

Our study suggests that immune responses to viral infections may be common underlying mechanisms for both diseases. Infections of viruses, such as herpes simplex virus (18), Epstein-Barr virus (19), and human papillomavirus (20), are risk factors for autoimmune uveitis in conditions such as Behçet’s Disease. Furthermore, the mechanism might not involve simple responses to viral replication but rather the long-term impact of viral infection on the immune system after the virus is no longer active (21). The correlation between insomnia and viral infections has also been extensively studied. For example, insufficient sleep in the week preceding symptom onset was associated with increased disease severity in patients with coronavirus disease 2019 (22). Conversely, the incidence of insomnia has been documented to significantly increase after viral infections (23). These studies indicate that insomnia and virus infection may mutually exacerbate each other, creating a positive feedback loop. Based on this, we hypothesized a link between viral infections and insomnia and uveitis. Long-term interaction between insomnia and viral infections may have effects on the immune system that increase the incidence of uveitis.

IFI44 and IRF9 regulate interferons, a family of cytokines with multiple effects in humans. First identified as antiviral inhibitors by Isaacs and Lindenmann in 1957 (24), interferons have since been recognized as crucial regulators of the human immune system (25). They play key roles in combating viral (26), and bacterial infections (27) and in autoimmune diseases (28). There are three types of interferons (IFNs): type I (IFN-α, β, ϵ, κ, and ω), type II (IFN-γ), and type III (IFN-λ), with signaling through the Janus kinase (JAK)/STAT pathway (29).

IFI44, identified in this study as a diagnostic biomarker for insomnia-associated uveitis, consists of 444 amino acids. IFI44 is an interferon-stimulated gene (ISG) induced by type I interferons, and it has diagnostic value in autoimmune diseases such as systemic lupus erythematosus and primary Sjögren’s syndrome. IFI44 is also associated with immune infiltrating cells and has been positively correlated with activated dendritic cells, immature B cells, and activated CD4+ memory T cells (30). T lymphocytes are considered to play a crucial role in the immunopathogenesis of uveitis (31). Type I interferons (e.g., IFN-α and IFN-β) play a key role in immune activation, thereby influencing the production and regulation of pro-inflammatory cytokines and other mediators (32). We hypothesize that the upregulation of IFI44 in patients with insomnia may promote the progression of uveitis by inducing innate immunity, activating acquired immunity, and modulating inflammatory cytokine and antibody levels.

IRF9 plays a role in the signaling of type I interferons (e.g., IFN-α and IFN-β) through the JAK-STAT pathway. When type I interferons bind to their receptors, they activate JAK kinases, which phosphorylate STAT1 and STAT2. Phosphorylated STAT1 and STAT2 heterodimerize and recruit IRF9 to form the ISGF3 complex, which enters the nucleus and activates the transcription of ISGs (33). In addition to mediating antiviral responses, IRF9 is also elevated in autoimmune diseases, such as systemic lupus erythematosus and rheumatoid arthritis. There is a substantial body of evidence supporting an important role of the IFN pathway in the pathogenesis of uveitis (32, 34). Moreover, novel agents targeting IFN-α significantly improve vision in uveitis patients. We speculate that elevated IRF9 in insomnia patients may promote the progression of uveitis by enhancing immune responses and the secretion of inflammatory cytokines. However, the specific mechanisms involved require further investigation.

The Protein kinase C (PKC) family consists of phospholipid-dependent serine/threonine kinases that are classified into three subfamilies based on their structural and activation characteristics: conventional/classical PKC isoforms (cPKCs: α, βI, βII, and γ), novel PKC isoforms (nPKCs: δ, ϵ, η, and θ), and atypical PKC isoforms (aPKCs: ζ, ι, and λ) (35, 36). PKCs are involved in various signal transduction pathways that control cell proliferation, differentiation, survival, invasion, migration, and apoptosis. In multiple sclerosis, a central nervous system inflammatory demyelinating disease, PKCβ inhibitors can stabilize the blood–brain barrier by targeting PKCβ in endothelial cells and possibly astrocytes, thereby inhibiting disease progression (37). In this study, PKCβ inhibitors also showed therapeutic potential in insomnia-associated uveitis.

Kenpaullone is an effective inhibitor of CDK1/2/5 and GSK3β. Cyclin-dependent kinase 2 (CDK2) is a negative regulator of Treg cell differentiation that is induced by transforming growth factor-β. CDK2 can directly phosphorylate FOXP3, disrupting its stability (38), and can inhibit Treg maintenance during inflammation (39). Inhibiting the GSK3β pathway can also promote Treg cell differentiation (40). Additionally, Treg cells (CD4^+^CD25^+^FOXP3^+^) are implicated in the development of Vogt-Koyanagi-Harada (VKH) disease (41). Therefore, Kenpaullone is a potential drug for insomnia-associated uveitis, but its efficacy and mechanism of action require further evaluation.

Our study identified the diagnostic significance of *IFI44* and *IRF9* in insomnia-associated AU; however, this study has some limitations. Firstly, the sample size was small. Future studies with larger sample sizes are needed to increase the statistical power and reliability of the results. Secondly, possible confounders of the data were not included in the matrix files (including sex, age, and age at onset) meaning that underlying bias was not controlled. Thirdly, the GSE66936 and GSE208668 datasets were derived from monocytes of autoimmune uveitis patients and from PBMCs of aged insomniacs, respectively. Therefore, it is hard to specify which cell type plays a major role in insomnia-associated uveitis by targeting *IFI44* and *IRF9*. Future research is needed to explore the precise mechanisms by which *IFI44* and *IRF9* contribute to insomnia-associated uveitis.

## 5 Conclusion

In summary, our study highlights the crucial role of interferons in both insomnia and uveitis, with a notable emphasis on the viral response. Additionally, further in-depth investigation of *IFI44* and *IRF9* involvement in insomnia and uveitis is warranted and *IFI44* and *IRF9* should be assessed as therapeutic targets.

## Data Availability

Publicly available datasets were analyzed in this study. This data can be found here: https://www.ncbi.nlm.nih.gov/geo/query/acc.cgi; https://www.ncbi.nlm.nih.gov/geo/query/acc.cgi;GEO; GSE66936, GSE208668.
